# Facies evaluation and sedimentary environments of the Yamama Formation in the Ratawi oil field, South Iraq

**DOI:** 10.1038/s41598-023-32342-9

**Published:** 2023-03-31

**Authors:** Israa A. Al-Iessa, Wang Zhi Zhang

**Affiliations:** Major of Geological Resources and Geological Engineering, College of Geosciences China University of Petroleum, Beijing, China

**Keywords:** Environmental sciences, Engineering, Materials science

## Abstract

Microfacies and environmental analyses of the Yamama Formation were conducted in the Tithonian–Hautervian sequence in the Ratawi oil field of Basra city in southern Iraq. The study includes petrographic, facies, and depositional models for the study area. Seven main facies and several secondary facies have been deposited in multiple environments. Moreover, they are affected by many diagenesis processes. Those facies have noncore depths that can be monitored by matching them with their corresponding well logs to obtain their electrofacies. The facies are distinguished according to the grain or mud supported or on the appearance of the configuration facies through microscopy. After comparing them with the well logs, electrofacies are identified as the following main limestone facies (mudstone, mudstone–wackestone, wackestone, wackestone–packstone, packstone, packstone–grainstone, and grainstone facies). The formation environments in this field are divided into several environments depending on the facies and electrofacies characteristics of the formation, including a lagoon environment, open marine environment, shoal environment, and slope environment.

## Introduction

The Yamama Formation is a heterogeneous reservoir that dates back to the Valanginian–Berriasian period in the Tithonian–Hautervian sequence^1^. This period witnessed the occurrence of complications, including the occurrence of wide and overlapping facies variation. It is characterized by common cyclic sedimentation in the facies sequence, reflecting a sedimentary environment and a specific geological age of the formation, which can be tabulated and followed vertically and horizontally within the section. As a result, the sedimentary model can be deduced, so current studies have tended to present the essential petrographic characteristics that characterized these components within the sedimentary section of the formation and then divide this section into several primary and secondary facies and distribute them within their environments according to the divisions of Wilson^2^. This study generates petrographic, facies analysis, building facies, and depositional environment models for the study area to determine the facies type distribution in each reservoir unit and its percentage in the study area.

## Geological setting

**Figure 1:**
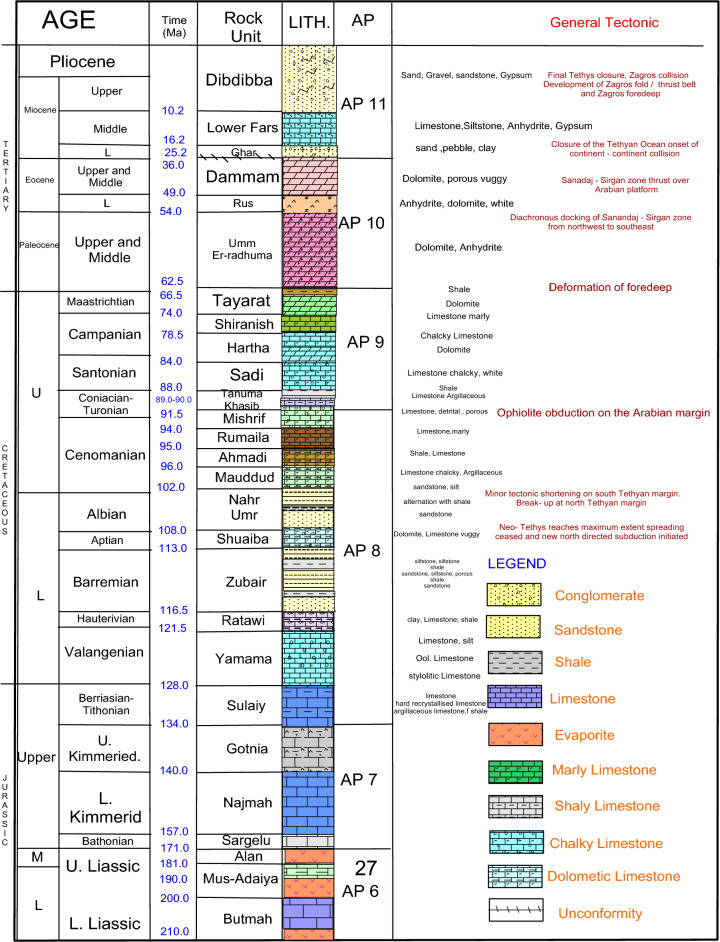
The generalized stratigraphic columnar section observed in the study area (generalized by using Didger 3 software).

One of the major reservoirs in the Ratawi oil field is the Yamama Formation, deposited at the foot of mega sequence AP8 (Tithonian–Early Turonian)^1^. The Yamama Formation was deposited in the Early Cretaceous and is laminar between the Sulaiy Formation in the lower transitional contact, which contains limestone, hard recrystallized limestone, and argillaceous limestone with occasional interbeds of shale. The Ratawi Formation is confined on top by clay and limestone with a significant amount of shale; see Fig. 1.

## Palaeogeography and Yamama Formation equivalents in the Middle East

Sharland et al., referred to tectonic events of the Arabian plate and surrounding masses that influenced reservoir, cover, and source sediment accumulation. The tectonic opening occurred between the Ptlas/Sargan plate during the end of the Jurassic and the Cretaceous^3^.

A passive margin, open to the sea, spans north and south of the Arabian plate. This powerful force drove oceanic crust spreading, resulting in faults that formed isolated rift basins of low energy and high continental crust edges of high energy, resulting in the construction of the carbonate ramp in northern Iraq represented by the Garagu basin. The Rayda basin to the south and southeast of the Arabian plate near the Oman Gulf resulted from localized subsidence of the fault blocks accompanying the entrance of the Indian Ocean^3^.

During the Berriasian–Valanginian period, with the conclusion of the Neo-Tethys Sea Formation, tectonic events at the end of the Jurassic period led to the construction of the Tikrit High, with the architecture extending from northwest to southeast. Due to erosion, a shallow basin grew deeper towards Amara, Halfaya, and Bazerkan, preparing it for the calcareous sediments of the Yamama basin.

**Figure 2:**
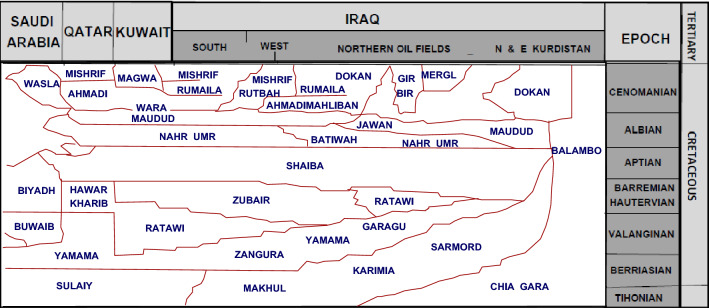
Equivalents to Yamama Formation in Iraq and the neighboring Arab countries modified from^8^ by using Didger 3 software.

**Figure 3:**
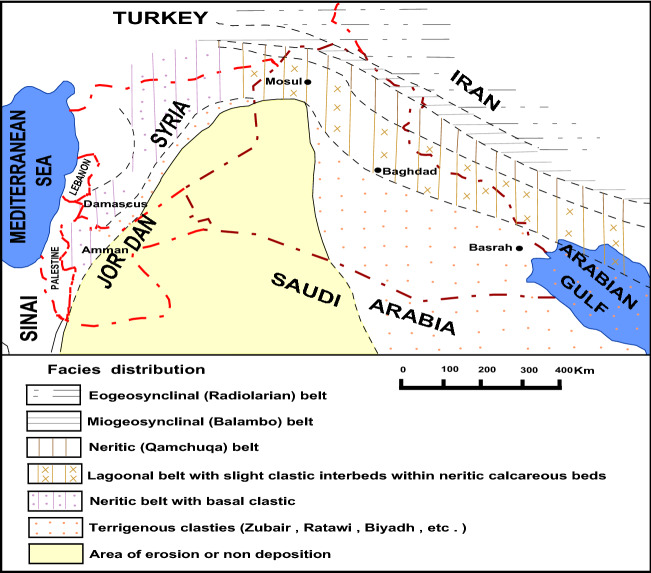
Paleogeography map of the Late Berriasian—Aptian period, modified form^4^ by using Didger 3. The miogeosynclinal furrow indicate that in northeastern Iraq the boundary of Balambo trough was formed by ridge indicated by shallow water carcareous sediments in the wider Surkev–Norbab–Avroman area. On the area of unstable shelf, and on the marginal parts of the stable shelf,—three zones, with different sedimentary sequences, can be distinguished in this figure: (**a**) The neritic belt, is relatively broad zone connected with the ridge separating the miogeosynclinal furrow from the basinal areas of the platform. (**b**) The lagoonal belt occupies the rest of the Foothill Zone, and the Mesopotamian Zone. The sediments of the basin are characterized by terrigenous supply, mainly in the basal parts of the sequence Garagu facies and show lagoonal in layers too. (**c**) The sandy zone occupies the rest of the Mesopotamian Zone (the Euphrates Block). The zone is characterized by prevalently terrigenous clastic sedimentation, pelitic and calcareous at the beginning i.e. by the reduced Yamama and the Ratawi Formations and mainly by the mighty Zubair sands. The area extends over the stable shelf on and to the east of the Abu Jir Subzone too.

"Transform faults" are faults that run north–south and crosswise in the direction of the Zagros, as seen by the continued sedimentation of the basin's Chia Gara Formation as the lower part. To the east is the Karimia Formation, a clay limestone with Berriasian rocks in its upper portions. In southern Iraq, the Sulaiy and Yamama Formations were deposited, and in the far east, the Palambo Formation, distinguished by Radiolaria fossils, was deposited. The Garagu Formation, one of the Yamama Formation counterparts, extends in the shape of a tongue within the Sarmord Formation^4^, as shown in Figs. 2 and 3.

## Study area

**Figure 4:**
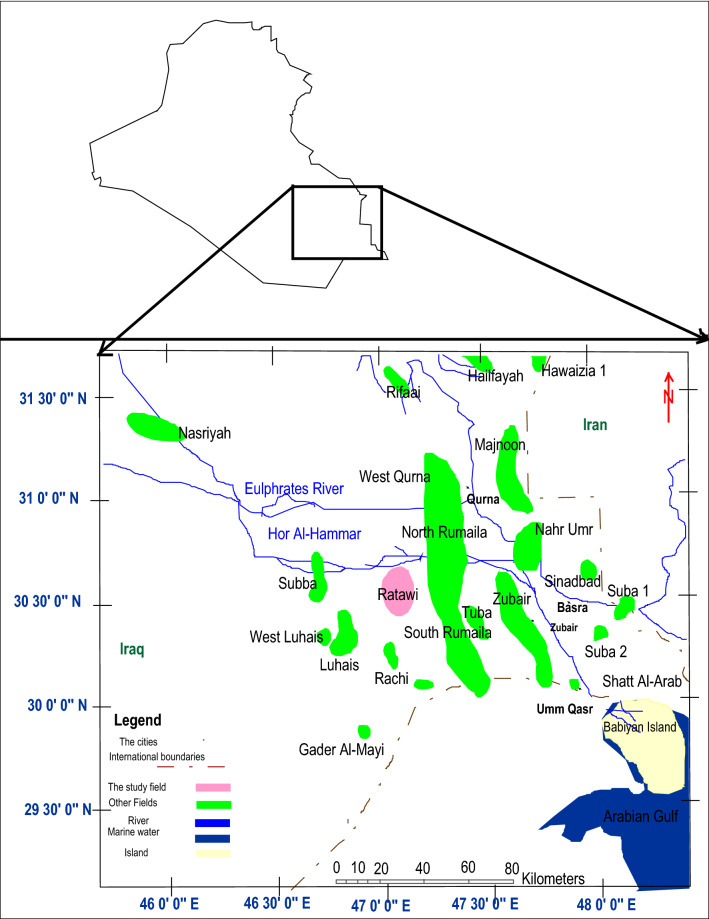
Location map of Ratawi oil field modified from map to Jassim and Goff^1^ by using Arc Map 10.3 UTM-WGS 1997, north 38.

The Ratawi Oil Field is located in southern Iraq, 70 kms west of Basra, 12 kms south of Hor Al-Hammar, and between latitudes (E 705.4-696.36) and (N 3394.183-3373.8) (Fig. 4). The Ratawi Field was identified by gravity surveys in early 1940 and afterwards by the Basra oil company in 1947–1948.

**Figure 5:**
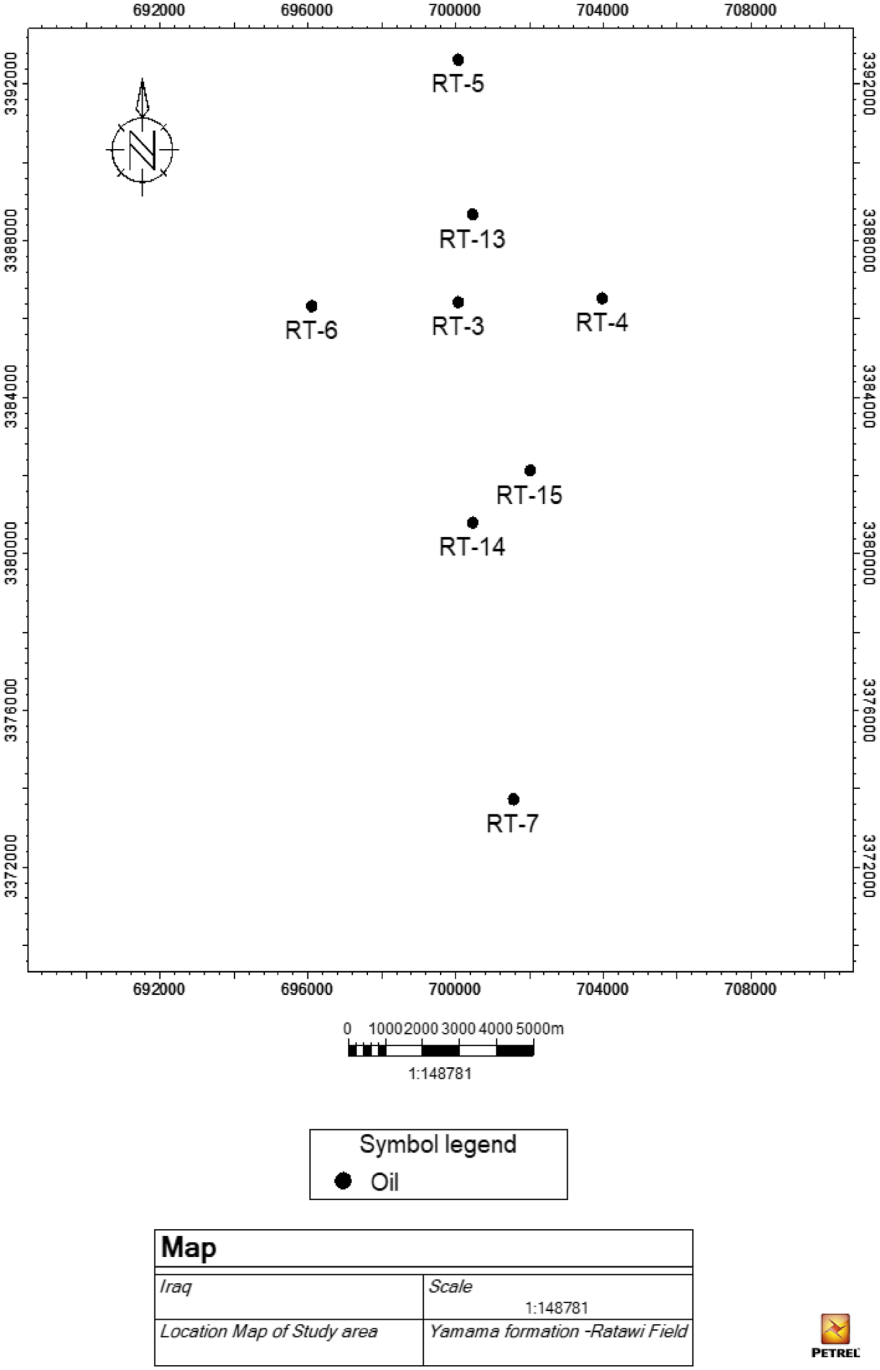
Location map of the study area with distributed of studied wells.

There are 28 wells in the field, nine of which penetrate the Yamama Formation. The availability of borehole sensors was adopted to select these wells distributed at the crest and flanks of the field; see Fig. 5. Eight wells were selected in the current study (Rt-3, Rt-4, Rt-5, Rt-6, Rt-7, Rt-13, Rt-14, and Rt-15).

## Methodology

1. Preliminary information was compiled and consisted of reviewing wells and research, including internal reports, geological and reservoir studies, and the archives of the Iraq Ministry of Oil and its services companies, along with information from the Oil and Gas University.
2. Wire-line logs were collected that cover most of the excellent section for Yamama wells, which were selected because of the availability of open-hole logs for identifying each reservoir unit in Yamama Formation, including the compensated neutron log (CNL), density log, sonic log, spontaneous potential log, resistivity logs, gamma-ray log, and calliper logs. These logs identify the formation tops and thickness and have also been used in stratigraphic correlation. These logs are also crucial for identifying the separator boundaries between the formations that return to the reservoir.
3. Detailed core examinations and thin sections were collected for facies analysis and diagenesis evaluation. Then, these components within the sedimentary section of the formation were divided into primary and secondary facies and distributed within their environments according to the divisions^2^ to draw sedimentary environment models for the study area. The steps for sampling and making a thin section are listed below:
  - Describing the study samples through field observations.
  - Modelling the study samples, photographing and describing the samples during collection, then storing them in special bags.
  - Making thin slides of the selected samples.
  - Examining the thin slides under a microscope and obtaining a clear picture of each important slide using a special microscope.
4. Upscaling the petrophysical parameters and facies results to build the facies models.
5. Applying software programs to obtain the following aspects:
  Didger 3 software was used to read the values of all open hole logs mentioned above with depth for each metre for the study area. Additionally, the program was used to draw many graphics and maps related to the study area.
  Excel was used to calculate the petrophysical properties of the reservoir.
  ArcMap, Photoshop, and Paint were used to draw many graphics and maps related to the study area.
  The Weka-3.6 program with Excel was used to determine the values of electrofacies for the study area to obtain the intervals of facies for the study area that had no core by open hole logs.
  The Petrel 2017 program was used as follows:
    - Tabulate the results of the petrophysical and facies analysis and make a correlation between the studied wells.
    - Make 2D and 3D models for the facies model after generating upscaling for these properties and make surface maps for the reservoir units.

## Previous studies

The Yamama Formation was described for the first time by Steinke and Bramkamp^5^ in Saudi Arabia, who indicated that it was one of the Al Thamama Formations, along with the Al Buwaib and Sulaiy Formations, which consisted of a fragmental limestone return back to the Lower Cretaceous period. While, Van et al.^6^ called it the Yamama–Sulaiy section in Well (Ratawi-1), which consisted mainly of different limestone sequences.

Both of Dunnington^7^ and Sadooni^8^ provided a description of the Yamama Formation combined with Sulaiy in Iraq. Based on this description, a composite formation (Yamama–Sulaiy) was selected as an example section of a (Ratawi-1) well in southern Iraq and a well (Burgan-113) in Kuwait represented by pelletal limestone located under the Ratawi Formation shale.

The Yamama Formation and Ratawi Formation have been redefined to confirm the rockslide difference between the two formations, which is represented by embodying the carbonate components to form Yamama mainly, while the Ratawi Formation represents mainly clastic rocks^9^. Calcareous algae in Yamama Formation sediments have been studied, and the formation age was estimated to be Valanginian in Siba-1^10^. While^11^, separated the Cretaceous epoch in Iraq into Upper and Lower Cretaceous, indicating that the Yamama and Ratawi strata include Tithonian–Late Berriasian fossils.

The Al-Yamama Formation has been studied regionally, indicating that most of the argillaceous and limestone rocks in the Al-Yamama Formation could be considered good source rocks^12^.

The Al-Yamama Formation has been studied based on its fossil content and is considered equivalent to the Zangura Formation (Valanginian–Berriasian) in northern Iraq, which consists of thick layers of limestone and calcareous clay–limestone for the first formation and calcareous rocks containing coral and algae for the second formation^4^.

The analysis of^13^ confirmed that the formation's upper and bottom limits are compatible and that the formation's ground is clay–limestone granules containing primarily algal particles. The Yamama Formation age was determined by Al-Abbadi^14^ by studying the stratification of life and temporal data (Late Berriasian–Valanginian).

The formation has been studied by stratigraphic methods showing that the Yamama sedimentation platform is a ramp platform^15^.

The rocks of the Yamama Formation in the West Qurna and Nasiriyah fields were deposited within two sedimentary phases and within two separate secondary basins^16^. However, Al-Shahwan^17^ confirmed that the formations of Sulaiy, Yamama, Ratawi, Zubair, and Nahr Umr are source rocks.

## Lithological units

**Figure 6:**
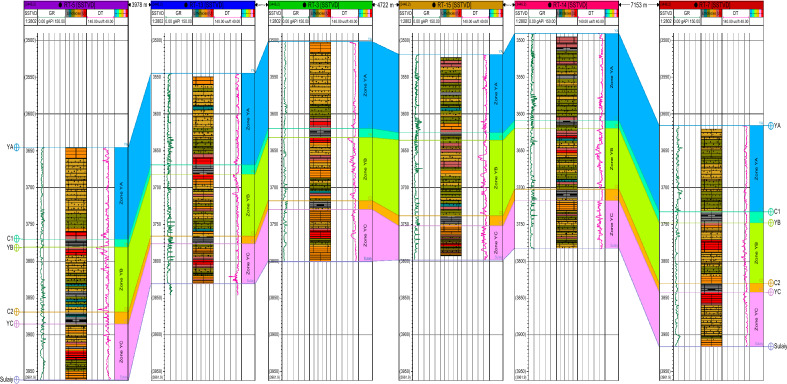
Correlation section in direction North–South passes through the study wells (Rt-5, Rt-13,Rt-3, Rt-15,Rt-14 and Rt-7).

**Figure 7:**
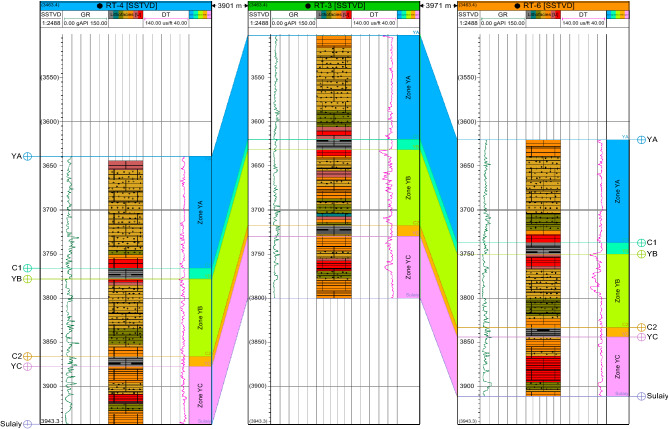
Correlation section in direction East–West passes through the study wells (Rt-4, Rt-3, and Rt-6).

The Yamama Formation is characterized by porous limestone interspersed with thin layers of argillaceous and tight limestone. Some shale strata revert to barrier units. They end in dense, compact limestone with a shaly layer in some wells and a uniform, gradient surface. The rarity and absence of fossils in the research wells set this limit. The lack of integrated core sections in the study area formation, the difficulty of studying cuttings due to challenges in obtaining particular or significant fossils compared to finer facies, the fear of mud contamination from drilling, and inaccuracies in determining model depths have made determining this limit by well logs onerous. To compare them with petrophysical parameters (porosity and permeability), the Yamama Formation was split into three primary reservoir rock units (YA, YB, and YC) (Fig. 7). (C1, C2) as barrier units^18,19^, see Figs. 6 and 7.

Each unit's lithology description is listed below:

- **YA unit:** This unit shows remarkable stability in thickness, as the average thicknesses were 118,126.87, 125.09, 116.93, 117.02, 124, 118.5 and 106.63 m. An examination of two well cores (Rt-5 and Rt-7) showed that it consists of grey limestone exchanged with a thin area of light brown limestone that contains some stylolite and little oil steaming, as well as interspersed with some thin layers from shale and compacted limestone.
- **C1 unit:** This unit represents the barrier unit between the two reservoir units YA and YB, as the average thicknesses of Rt-3, Rt-4, Rt-5, Rt-6, Rt-7, RT-13, RT-14, and RT-15 are approximately 12, 12.33, 10.51, 12.8, 14.98, 13, 10.5, and 9.73 m, respectively, containing argillaceous limestone interspersed with thin layers of shale.
- **YB unit:** This unit is considered one of the most critical units of the Yamama Formation in terms of reservoir properties, and the average thicknesses are 86, 87.8, 88.01, 83.27, 81.97, 84, 82.5, and 102.77 m. It can be divided into two parts, upper and lower: the upper part of it, made of light brown limestone contains a little bit of show oil, along with some stylolite lines, while the lower part has less important reservoir quality specifications than the previous one, as it is noted that the percentage of shale (Vsh) has increased more than the upper part within this unit.
- **C2 unit:** Depending on the examination of the available core, this unit represents the second main insulating layer between the two reservoir units YB and YC, as it is composed of argillaceous limestone exchanged with layers of shale. The average thicknesses of Rt-3, Rt-4, Rt-5, Rt-6, Rt-7, RT-13, RT-14, and RT-15 are 12, 11.57, 16.39, 10.73, 12.06, 10.5, 15.5, and 13.94 m, respectively.
- The **YC unit** consists of grey limestone containing some stylolite and some oil shows, and the average thicknesses of Rt-3, Rt-4, Rt-5, Rt-6, RT-7 RT-13, RT-14, and RT-15 are 70, 65.38, 76, 67.36., 73.97, 53.92, 64.58, and 46.04 m, respectively.

This formation lies between the Sulaiy Formation in the lower transitional contact, containing argillaceous limestone with some shale, and the Ratawi Formation, bounded on top by clay and limestone with a high percentage of shale. This means that the Yamama Formation is divided into three main lithofacies zones, the first representing rocks of wacky and pellet packstone and grainstone of limestone with good porosity and permeability at the lower parts of wells located on the crest of the field, the second representing a mix of packstone, oolitic, and peloidal grainstone of limestone with some intervals of wacky limestone deposits in the middle of the formation, whereas the third lithofacies deposits at the upper part of the formation represent a mix of oolitic and grained limestone and different kinds of wacky limestone, with some intervals of layers containing clay limestone in some wells towards the southern area of the field. Thus, the Yamama Formation is divided into:

## Petrographic constituents of microfacies

There are numerous limestone facies classes, each with distinct properties. In the current study, Dunham's categorization^20^ was used to classify the microfacies based on the sedimentary environment, rock texture, grain quality, and agglomeration. This categorization divides limestone rocks into two basic types: grain supported and mud supported. These petrographic components (grains, groundmass, or matrix) are described as follows:

1. **Matrix or groundmass:**
  The groundmass is one of the essential indications of depositional energy intensity and is made up of micrite and sparite depending on the particle size^21^. The groundmass in this investigation was micrite partially converted into microsparite by cementation or neomorphism. Micrite is the substance that fills the gaps and compartments of fossils. In some research regions, the micrite partially transformed into sparite, leaving pores within the ground as a result of exposure to dissolving processes. Dolomitization altered the groundmass, as seen in the Yamama Formation rocks by the diffusion of rhomboid dolomite crystals in some microfacies.
2. **Grains:**
  Grains are particles that are precipitated mechanically and originate before sedimentation or through it. Grains in carbonate (limestone) rocks divide into two types: skeletal grains and nonskeletal grains^22^. The following is a review of the essential grain constituents observed in the limestone of the Yamama Formation.
  Skeletal grains: It was found by examining the thin sections that the wells in the Yamama Formation contained a percentage of skeletal grains, which include calcareous algae, some larger benthonic foraminifera such as Everticyclamina eccentric and Pseudocyclammina littus, some smaller benthonic foraminifera such as Cyclammina greige, Nautiloculina Politica, and Trocholina Alpina, echinoids, and a few sponge spicules and molluscs, which can distinguish the study area by containing an abundance of these skeletal grains.
  Nonskeletal grains: The nonskeletal grains that were distinguished in the facies of the study area were represented by oolites, pseudoolites, and peloids, and some pellets were also observed very rarely.

## Microfacies analysis

Many studies have used different definitions of microfacies, but Flugel^23^ used a simplified and straightforward definition, which is the sum of sedimentary and fossil features that may be defined and classified through the thin section. According to Kendall, sedimentary deposits' textural, structural, and compositional features result from deposition and change in a specific sedimentary environment^24^. More than 250 thin sections of the Yamama Formation were studied in three Ratawi oilfield wells (Rt-3, Rt-5, and Rt-7) from north to south. The facies for the intervals that do not have cores was electrofacies utilizing well logs (∆t, ∅N, RHOB, SP, and GR), thus, seven types of main microfacies were identified based on the existence of grains to the groundmass, and the critical diagenesis effect on those rocks was defined based on Dunham^20^. Secondary microfacies (submicrofacies) are defined by the fossils and grains that indicate the sedimentary settings. The microfacies were compared to Wilson standard microfacies (SMF)^2^ and the environment to environmental facies zones (EFZs)^22^.

1. **Lime mudstone main microfacies**
  At the base of the micritic facies^20^, classified lime mudstone as a form of limestone whose primary structure is composed of microcrystalline calcite, which corresponds to the name (micrite) coined by Folk^19^. This facies was affected by many diagenesis processes, mainly dolomitization and recrystallization, followed by compaction, micritization, dissolution, and plate formation (1–1, 1–2, and 1–3). Compaction affected the emergence of pressure solution (stylolite) in some study wells. It was noted that dolomitization affects these main facies differently. First, early dolomitization is inferred from xenotropic dolomite (aphanotopic texture), and late dolomitization is represented by coarse dolomite with large crystals. Recrystallization or neomorphism impacts micrite, turning it into microsparite or pesudosparite, which changes facies components. This main facies has intercrystalline, vuggy, and mouldic porosity from washing and dissolving and reduced porosity from cementation with granular and spare cement. There are also some authigenic mineral formations (pyrite) and bitumen traces. These major facies were found in all of the study wells, mainly in the barrier units of the research well sections, with sporadic appearances in the middle, upper, and lower parts. This facies is deposited in an organic clay environment.
2. **Lime mudstone—bioclastic wackestone main microfacies**
  These main facies overlap between two facies: lime mudstone facies with bioclastic wackestone facies and contains green and red algae, debris of smaller benthonic foraminifera, and a few mollusc shells that may be greater or less than (10%) within micrite groundmass. Where it can be classified depending on Folk^25^, it is fossiliferous micrite to spare biomicrite, and this main facies indicates a transitional environment between shallow open platforms or shelf lagoon environments within facies zone (FZ-7) and restricted platform environments within facies zone (FZ-8) according to Wilson's^2^ divisions of the standard region,see Plate (1–4).
3. **Wackestone main microfacies**
  These major facies are found in several of the current study wells. The skeletal grains found are benthonic foraminifera, echinoids, mollusca, and algae. Peloids and some bioclastic detritus are nonskeletal grains. These principal facies have been impacted by diagenesis processes such as dissolution, micritization, compaction, dolomitization, and cementation to varying degrees, depending on the environmental circumstances at the time of sedimentation. Porosity decreased at some depth intervals due to cementation and void filling; see Plates (1–5), (1–6), (2–1) and (2–2).
4. **Wackestone–packstone main microfacies**
  This main facies is characterized by an increase in the percentage of skeletal grains, and it consists of bioclastic algae debris with a small percentage of mollusc shells and echinodermata, very little coral debris, and some benthonic foraminifera pieces such as miliolipids, where the percentage of grains in this main facies ranges from 40 to 70% compared to groundmass. Grains appeared in different parts of the study formation and at different rates from one well to another; see Plate (2–3).
5. **Packstone main microfacies**
  These main microfacies are characterized by a granular ratio of 70–90% represented by peloidal, pseudoolitic, oolitic, some pellets, and some different kinds of bioclastic debris and fragments within micrite groundmass. They were wholly or partially transformed into microsparite or pseudosparite by neomorphism process compartments of some fossils, voids, and solution microchannels filled with spary cement by dissolution and cementation processes. The dolomitization process also affects them, consisting of acceptable to medium-sized rhomboid crystals distributed in different ways from one well to another within the groundmass of these main facies. This main facies appeared in most of the study wells and in all parts of the formation. It varies from one well to another and is divided according to the type of granules prevailing in them; see Plates (2–4), (2–5) and (2–6).
6. **Peloidal packstone–grainstone main microfacies**
  These main facies consists of a clear overlap between peloidal packstone microfacies, along with some benthonic foraminifera and echinoids, and grainstone microfacies. This main facies is characterized by the abundance of grains in terms of quantity and size. They may reach the size of small pebbles with sculpted edges (abraded) due to the high energy for the sedimentation environment with a percentage of micrite groundmass remaining. It was noted that it was affected by the washing process, or dissolution, consisting of vuggy, mouldic, and intergranular porosity. However, the cementation process led to the filling of voids and reduced the percentage of porosity. For the dolomitization process, its effect is small, as dolomite appears in a small percentage in this main facies. These main facies can be located between a biostrome environment or the so-called shallow barrier environment. Additionally, an open shallow lagoon environment is roughly part of the standard facies (SMF-15) of the facies zone (FZ-6) and the standard facies (SMF-17) from the facies zone (FZ-8) according to Wilson^2^ for standard facies, see Plate (3–1).
7. **Grainstone main microfacies**
  In total, skeletal grains and nonskeletal grains make up more than 90% of the basic structure of these main facies, along with less than 10% of the groundmass, which is often composed of microsparite or pseudosparite. These main facies are affected by different diagenesis processes, such as cementation, neomorphism, and the dolomitization process; see Plates (3–2) and (2–3).

## Electrofacies

Each environment has a regular series of electrofacies patterns described as a group of log responses reflecting features and facies qualities^26^. Logging is now widely used in subsurface geological studies to compensate for the lack of core samples. Thus, these facies investigations fill in the gaps. This study used well logs both quantitatively and qualitatively. The qualitative explanation compares the rocky facies and microfacies of wells that have no core samples or rocky slides by well logs to the available rocky facies and microfacies of wells that do have core samples or rocky slides. Quantitative interpretation is made by electrically comparing layers and water with layers, calculating porosity, water saturation, shale distribution ratios, etc.

This study was used to determine the electrofacies by the response of open well logs (Formation Density Compensated, Gamma Ray Log, CNL Log—Neutron Compensated, and Sonic log or Borehole Compensated—BHC Log) with core data. The information above was used in Weka software to predict facies for interval depths and then compare them with the results of upscaling with specific geostatistical methods in Petrel petroleum software to obtain the most realistic results for facies for all interval depths for the wells in the study area.

## Depositional environments

The depositional environment is defined as all the physical, chemical, and biological conditions in which sediments accumulate. It is believed that approximately 90% of the carbon deposits present in the environments are of biological origin and were formed under marine conditions^2,27,28^. To determine the sedimentary environments, it is necessary to know and diagnose the microfacies, identify their most essential components, and then compare them with the standard facies of Wilson^2^ and secondary environments defined by Flugel^22^. Therefore, the depositional environments for the Yamama Formation in the Ratawi field were determined as follows:

1. **Lagoon environment**
  This environment includes lime mudstone main microfacies. Some contain lime mudstone—bioclastic wackestone main microfacies. Benthonic foraminifera wackestone submicrofacies, green algal wackestone—packstone submicrofacies, some pellet packstone submicrofacies, peloidal packstone submicrofacies, and some peloidal packstone—grainstone main microfacies within facies zone (FZ-8) represent precipitation in the tidal flats that are restricted. They have protected lagoon environments. The essential characteristics of these facies are that they are deposited in a relatively shallow environment and have subtle sedimentary energy and limited movement. The microfacies of this environment are within the standard facies zone of Wilson^2^ (FZ-8).
2. **Open marine environment**
  This environment includes some lime mudstone—bioclastic wackestone main microfacies, bioclastic wackestone submicrofacies, corallian algal wackestone submicrofacies, some red algal wackestone—packstone submicrofacies, some pellet packstone submicrofacies, and some oolitic and pseudooolitic packstone submicrofacies. These microfacies are deposited in shallow water with open circulation—open platform or shelf lagoon environments, within the standard facies zone of Wilson (Jassim and Goff^1^, (FZ-7).
3. **Shoal environment**
  The external shape depends on the energy of the waves and the intensity of the sea storms. Thus, the barriers for these deposits are numerous and of enormous size due to the high energy. Therefore, these microfacies are deposited in the shallow barrier and shallow shoal environments within the standard facies zone of Wilson^2^ (FZ-6). This environment includes some oolitic and pseudooolitic packstone submicrofacies, some peloidal packstone—grainstone main microfacies, oolitic and pseudooolitic grainstone submicrofacies, and peloidal grainstone submicrofacies, as longitudinal barriers are formed in the environment submerged underwater.
4. **Slope environment**
  This environment includes bioclastic wackestone—packstone submicrofacies. These microfacies represent the foreslope environment because of packstone predominance over wackestone within the standard facies zone of Wilson^2^ (FZ-4).

## Depositional model

As a theoretical background for the depositional model, Al-Hamdani^29^ stated that a good sedimentary model summarizes and reflects all environmental impacts detected during the interpretation and study of microfacies.

The geological model of the research area is a carbonate ramp per^30^ and^31^. It is an inclined platform extending from the high-energy shallow coastal environment to the deep environment^2^. As coastal structures develop in high-energy environments, carbonate ramps are an early developmental stage for establishing the carbonate shelf edge. According to Tucker et al.^28^, a sudden and severe change in tilt occurs when we approach the deep waters of the carbonate shelf.

Second-order sequence #1. Because the homoclinic ramp indicates a sedimentary lime system for a ramp platform with a modest incline, the facies distribution of Yamama Formation rocks in the research area shows this^30^. As indicated by Sharland^3^, the stratigraphic comparison of the Yamama Formation with global sea-level change confirms the formation deposition. From the top of the Sulaiy component of the highstand system track, where the Yamama Formation begins to precipitate, to the bottom of the Ratawi Formation, it represents one considerable, regressive sedimentary period. This is one of the three second-order sequences that made up the Lower Cretaceous period^32^.

**Figure 8:**
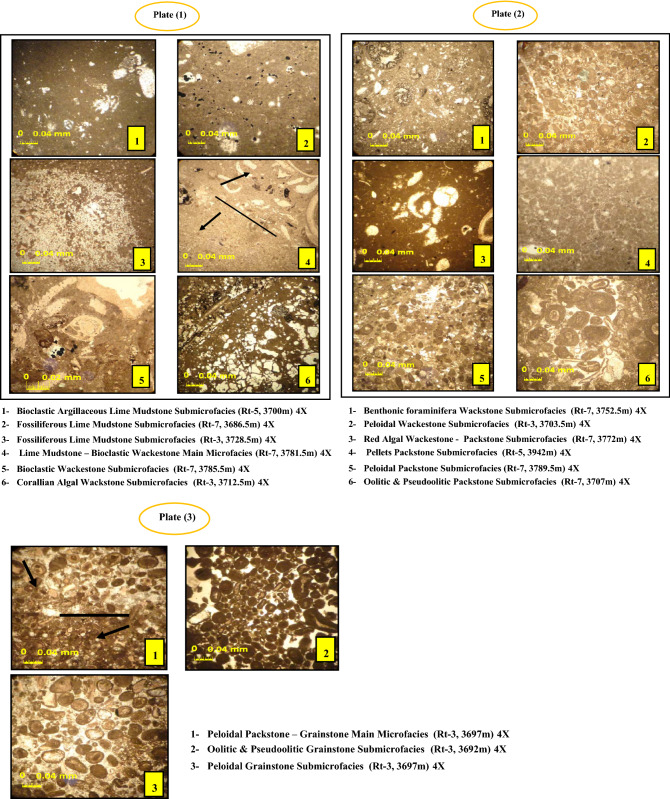

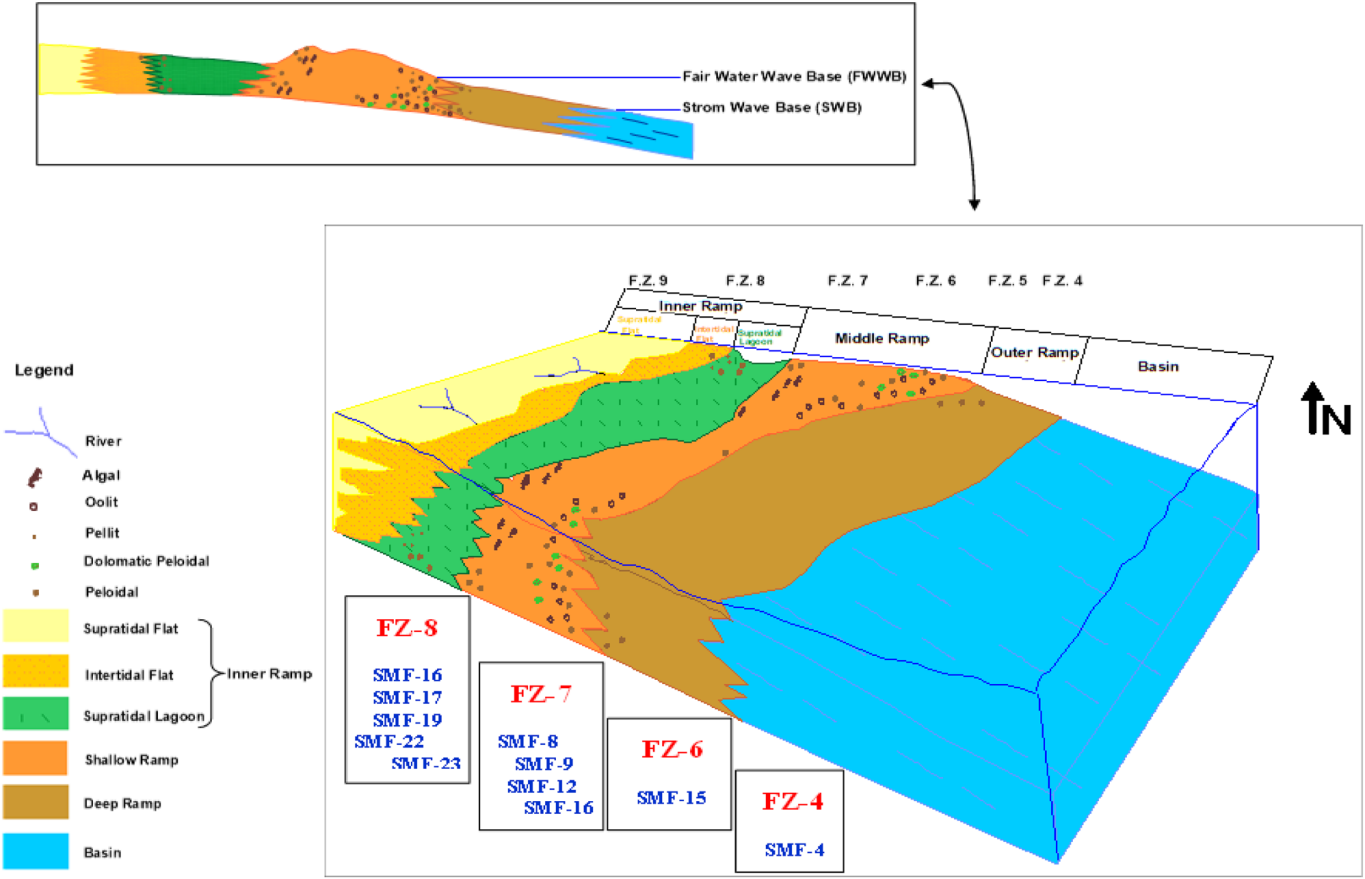
The proposed sedimentary model for Yamama Formation in Ratawi field for the current study by using Didger 3 software for drawing the graphic and compare it to the main ideas references that have been mentioned in the depositional model. So, the current study' proposed sedimentary model was based on the typical microfacies distribution model of Wilson^2^ and the sedimentary model of the carbonate ramp proposed by Ahr^30^ and Read^31^. The final form will be as seen in Fig.  8. Has been added author legend depending on spreading author petrographic constituents of microfacies. Also, it has been put the related Wilson standard microfacies (SMF) depending on author main microfacies and submicrofacies distribution in the model, and the related environmental facies zones (EFZ) depending on author main microfacies and submicrofacies environments. Modified the ideas of the distribution related to each one exactly to match it depending on author model sample and type.

When the environment and conditions of each facies are diagnosed, it becomes easier to determine the sedimentation environment and conditions of each facies diagnosed due to the interpretation of the sea level in the area. For the Yamama Formation, the current study's proposed sedimentary model was based on the typical microfacies distribution model of Wilson^2^ and the sedimentary model of the carbonate ramp proposed by^30^ in JOGMEC^33^. The final form can be seen in Fig. 8.

## Porosity types in Yamama rocks

Porosity is described as a measure of what the rock holds in terms of voids and pores that carry reservoir fluids as one of the most critical reservoir features^28^, and porosity in limestone is usually not homogeneous. It has a different origin than clastic rocks due to diagenesis processes affecting limestones, such as dissolution, fracturing, dolomitization, and cementation, which can change the size and quantity of pores in limestone. Porosity is classified into two categories based on its creation stages:

1. Primary porosity: produced at the end of the sedimentation process.
2. Secondary porosity: produced after sedimentation and due to diagenesis processes.

Porosity in limestone is classified by pore type and origin, along with the relationship between rock type and pore type. The most typically utilized classifications in investigations are from^34^. The porosities that have been observed in the Yamama Formation of sediments are interparticle porosity and vuggy porosity, such as mouldic porosity, intrafossil porosity, solution enlarged fractures, and fracture porosity, depending on the geological and petrographic Lucia classification of pore types^35^.

## Facies distribution

**Figure 9:**
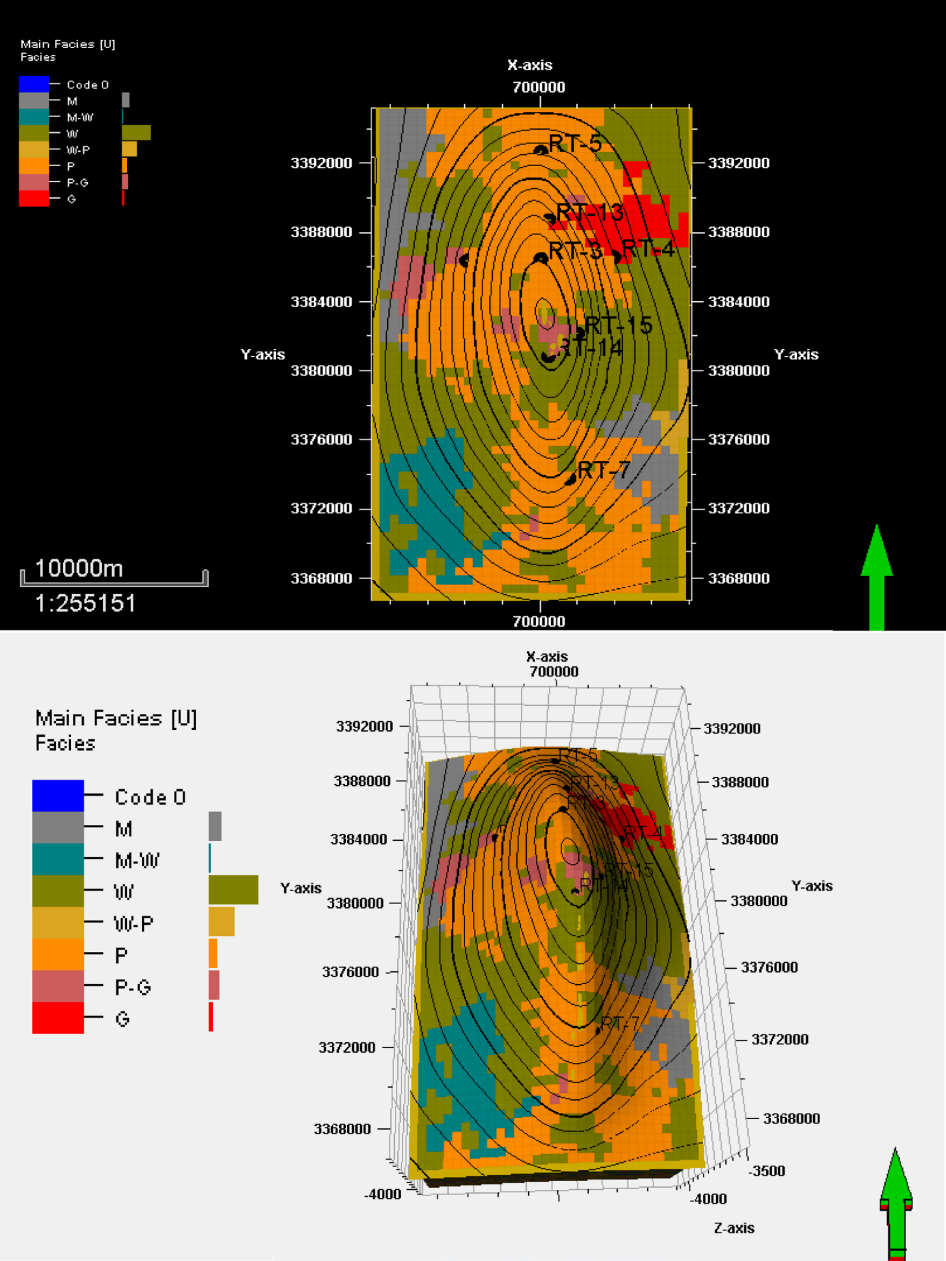
Facies distribution of the top surface of reservoir unit YA.

**Figure 10:**
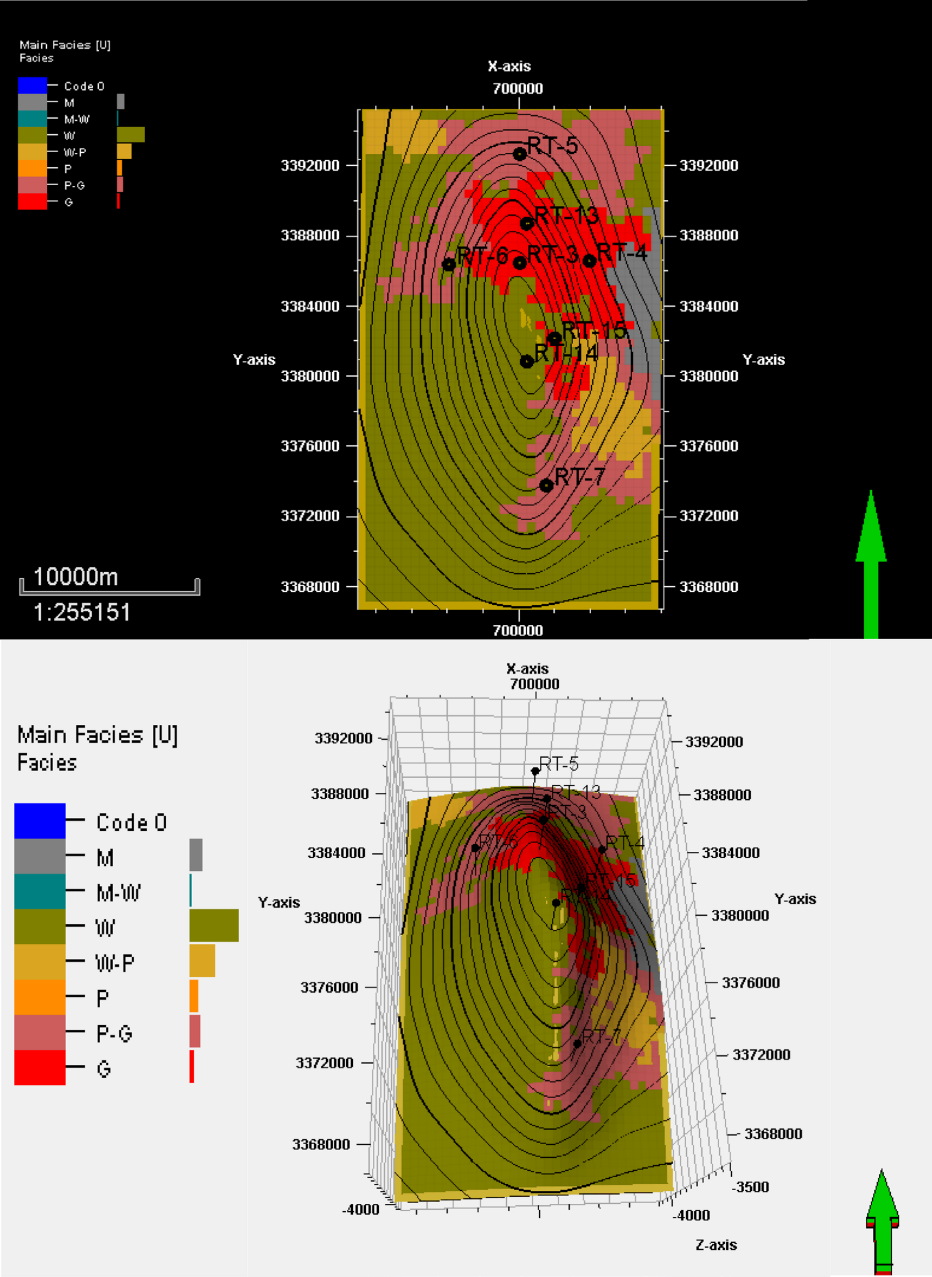
Facies distribution of the top surface of reservoir unit YB.

**Figure 11:**
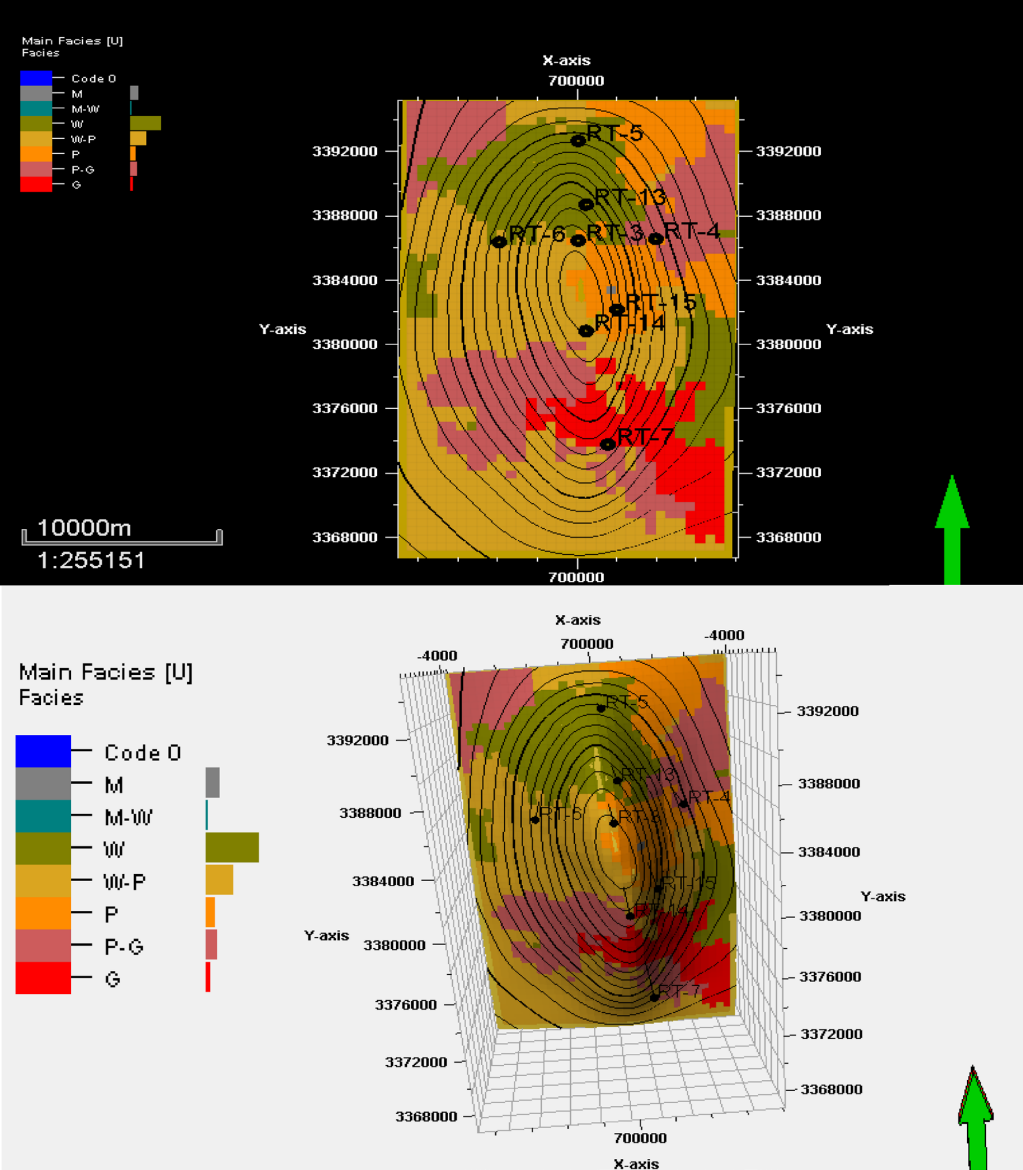
Facies distribution of the top surface of reservoir unit YC.

After explaining the main microfacies types and making electrofacies for the intervals that have no core samples using Weka and Petrel software, the percentage of each main microfacies that can reflect a specific sedimentary environment and its distribution in each reservoir unit are determined in Table 1, and the facies distributions of the top surface of reservoir units YA, YB, and YC are noted in Figs. 9, 10, and 11.

**Table 1:** Facies distribution of reservoir units of Yamama Formation.

Reservoir Unit YA	M (%)	M–W (%)	W (%)	W–P (%)	P (%)	P–G (%)	G (%)
RT-3	0	0	16	60	13	8	3
RT-4	0	0	4	68	4	12	12
RT-5	0	0	16	60	17	3	4
RT-6	0	0	16	56	21	0	7
RT-7	0	0	51	44	5	0	0
RT-13	0	4	24	52	8	4	8
RT-14	9	0	56	16	0	19	0
RT-15	4	4	48	20	8	16	0

Reservoir Unit YB	M (%)	M–W (%)	W (%)	W–P (%)	P (%)	P–G (%)	G (%)
RT-3	0	4	20	28	27	13	8
RT-4	0	0	21	60	11	3	5
RT-5	8	14	24	42	0	4	8
RT-6	0	0	25	45	11	7	12
RT-7	0	0	12	48	20	4	16
RT-13	0	4	29	35	16	8	8
RT-14	0	8	44	20	4	16	8
RT-15	0	4	44	28	4	20	0

Reservoir Unit YC	M (%)	M–W (%)	W (%)	W–P (%)	P (%)	P–G (%)	G (%)
RT-3	0	0	14	14	52	4	16
RT-4	0	0	11	20	53	4	12
RT-5	4	0	24	24	27	4	17
RT-6	0	0	12	15	28	0	45
RT-7	0	0	4	60	12	4	20
RT-13	0	0	24	16	28	20	12
RT-14	0	0	36	40	16	8	0
RT-15	0	0	19	33	28	12	8

M: Mudstone, M–W: Mudstone–Wackestone, W: Wackestone, P: Packstone, P–G: Packstone–Grainstone, G: Grainstone.

## Discussion

1. The Yamama Formation was divided into three central reservoir units (YA, YB, and YC) and two central barrier units (C1 and C2). The boundary of the contact between the upper and lower Yamama Formation of the Ratawi field was redefined using well logs, facies, and petrophysical data. Thus, the various reservoir data and new limits were given according to the data of the previous results, and these limits came very close to the results obtained from the studies of the oil service company JOGMEC^36^.
2. Formación Yamama differences and diverse distributions of these facies or the effect of formation rocks have established a complex system of facies in the Ratawi field. The most crucial diagenesis processes are dissolution and cementation, together with micritization, compaction, and dolomitization.
3. Those at noncore depths could be monitored by matching them with their corresponding well logs to obtain their electrofacies. The main facies were distinguished depending on whether they were grain supported or mud supported and on the appearance of the configuration facies through microscopy and after comparison. A variety of habitats were identified in the Ratawi field based on facies and the electrofacies features of the Yamama Formation: lagoon environment, open marine environment, shoal environment, and slope environment.

## Conclusions

1. The boundary of the contact between the upper and lower Yamama Formation of the Ratawi field, as well as the internal boundaries, have been redefined using well log data, facies data, and various reservoir data, and new limits were given according to the data of the results mentioned earlier.
2. It was found by examining the thin sections that the wells in the Yamama Formation contained a percentage of skeletal grains, which include calcareous algae, some benthonic foraminifera, echinoids, and a few sponge spicules and molluscs, which can distinguish the study area by containing an abundance of these skeletal grains. While the nonskeletal grains were represented by oolites, pseudoolites, and peloids, some pellets were rarely observed.
3. Seven primary facies and several subsidiary facies were found, deposited in various settings and modified by diagenesis. Facies at noncore depths could be checked by comparing them to their well logs. After comparing the microscopy results with the well logs, the following main limestone facies were identified: mudstone, mudstone–wackestone, wackestone, wackestone–packstone, packstone, packstone–grainstone, and grainstone facies.
4. The Yamama Formation environments in the Ratawi field were divided into several environments depending on the facies and electrofacies characteristics of the formation (lagoon environment, open marine environment, shoal environment, and slope environment).

## Data Availability

'The datasets generated and/or analyzed during the current study are not publicly available because the data is oil field data, not normal data, so just the final schedules, the plates and the figures are allowed for public but not the initial one or the details. So, the data that support the findings of this study are available from the corresponding author but restrictions apply to the availability of these data, which were used under license for the current study, and so are not publicly available. The final version of data is however available from the corresponding author.' in "Data Availability" section of this manuscript.
